# A ternary SnS_1.26_Se_0.76_ alloy for flexible broadband photodetectors†

**DOI:** 10.1039/c9ra01734h

**Published:** 2019-05-07

**Authors:** Lena Du, Cong Wang, Jingzhi Fang, Bin Wei, Wenqi Xiong, Xiaoting Wang, Lijun Ma, Xiaofeng Wang, Zhongming Wei, Congxin Xia, Jingbo Li, Zhongchang Wang, Xinzheng Zhang, Qian Liu

**Affiliations:** CAS Key Laboratory of Nanosystem and Hierarchical Fabrication, CAS Center for Excellence in Nanoscience, National Center for Nanoscience and Technology Beijing 100190 P. R. China; University of Chinese Academy of Sciences Beijing 100049 P. R. China liuq@nanoctr.cn; The MOE Key Laboratory of Weak-Light Nonlinear Photonics, TEDA Institute of Applied Physics, Nankai University Tianjin 300457 P. R. China zxz@nankai.edu.cn; State Key Laboratory of Superlattices and Microstructures, Institute of Semiconductors, Chinese Academy of Sciences Beijing 100083 P. R. China jbli@semi.ac.cn; Department of Quantum and Energy Materials, International Iberian Nanotechnology Laboratory (INL) Avenida Mestre José; Veiga s/n Braga 4715-330 Portugal zhongchang.wang@inl.int; Department of Physics, Henan Normal University Xinxiang 453007 P. R. China

## Abstract

Layered two-dimensional (2D) materials often display unique functionalities for flexible 2D optoelectronic device applications involving natural flexibility and tunable bandgap by bandgap engineering. Composition manipulation by alloying of these 2D materials represents an effective way in fulfilling bandgap engineering, which is particularly true for SnS_2*x*_Se_2(1−*x*)_ alloys showing a continuous bandgap modulation from 2.1 eV for SnS_2_ to 1.0 eV for SnSe_2_. Here, we report that a ternary SnS_1.26_Se_0.76_ alloy nanosheet can serve as an efficient flexible photodetector, possessing excellent mechanical durability, reproducibility, and high photosensitivity. The photodetectors show a broad spectrum detection ranging from visible to near infrared (NIR) light. These findings demonstrate that the ternary SnS_1.26_Se_0.76_ alloy can act as a promising 2D material for flexible and wearable optoelectronic devices.

## Abstract 2

Bandgap engineering of a ternary SnS_1.26_Se_0.76_ alloy for flexible broadband photodetectors.

## Graphical abstract

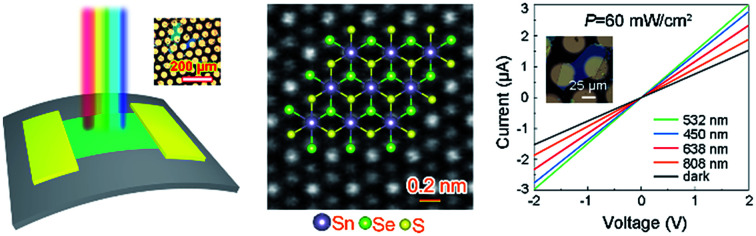

## 1 Introduction

Flexible optoelectronic devices that can be bent, stretched, twisted and folded have triggered wide interest due to their greater superiorities in flexibility, lightweight and transparency,^1–3^ and thus have potential applications in flexible solar cells,^4^ video imaging,^5^ optical fiber communication.^6^ muscle-like transducers,^7^ machine vision,^8^ and so forth. Layered 2D materials are naturally suitable for various thin-film (opto)electronic devices due to their ultrathin thickness and flexibility.^9,10^ Although graphene has excellent mechanical^11^ and (opto)electronic^12^ properties and can offer a series of new possibilities at reduced dimensionality, it is limited to application in photoelectronic devices due to its gapless nature.^13^ As an alternative, several 2D materials with sizable bandgaps and superior (opto)electronic performances^14–16^ have been considered as potential candidates for next-generation ultrathin and flexible photoelectronics.^17^ For example, significant attention has been devoted to monolayer MoS_2_, which exhibits a photoresponsivity as high as 5.75 × 10^3^ A W^−1^ at a bias voltage of 5 V.^18^ The carrier mobility of MoS_2_ at extremely low temperature can be as high as 34 000 cm^2^ V^−1^ s^−1^,^19^ and could be increased by strain engineering.^20^ In addition to the choice of materials, bandgap engineering^21,22^ enables to the modulation of electronic structure of 2D materials (*e.g.* indirect-to-direct transition), thus manipulating their optical and electronic properties. To date, alloying 2D materials has been demonstrated to be an effective way in fulfilling bandgap engineering for many optoelectronic applications.^23,24^

As environmental-friendly, reserve-abundant and low-cost materials, layered tin dichalcogenides (SnS_2_, SnSe_2_) are desirable for sustainable development of flexible optoelectronic devices. Alloying of SnS_2_ and SnSe_2_ is often effective for bandgap engineering, in which a continuously tunable bandgap of SnS_2*x*_Se_2(1−*x*)_ (0 < *x* < 1) from 2.1 eV (SnS_2_) to 1.0 eV (SnSe_2_) could be achieved.^25^ The layered SnS_2_ has a rapid photoresponsive feature with response time as short as 5 μs,^26^ and 1T atomic layered SnSe_2_ with a very close bandgap to silicon (1.1 eV) displays a good responsivity of 0.5 A W^−1^ and a fast response time of 2.2 ± 0.3 ms.^27^ Previous reports indicated that physical properties of a ternary compound SnS_2*x*_Se_2(1−*x*)_ can be tunable by varying the atomic composition *x*, giving rise to remarkable (opto)electronic properties that may not appear in either of its binary compounds.^28,29^ As a member of SnS_2*x*_Se_2(1−*x*)_ alloy, few layered SnS_1.26_Se_0.76_ (*x* ∼ 0.6) with a hexagonal CdI_2_-type structure in space group *P*3̄*m*1 is easily obtained by mechanical exfoliation from a crystal due to weak van der Waals force among layers. This low-dimensional semiconductor nanosheets with applicable bandgap represents a typical candidate for flexible optoelectronic devices due to its excellent optoelectronic properties possibly for broad spectra detection. However, the reports so far on the SnS_1.26_Se_0.76_ nanosheet-based flexible optoelectronic devices are very scarce, especially in the aspects of its mechanical durability, reproducibility, and high photo-sensitivity. In this work, we investigate flexible photodetectors based on ternary SnS_1.26_Se_0.76_ nanosheets, and demonstrate that the photodetectors exhibit a high photoresponsivity of ∼262 A W^−1^, a high detectivity of 1.98 × 10^11^ jones and a fast response time of ∼10 ms to 532 nm light. Interestingly, the devices display a broad spectra response ranging from visible to NIR light and an ultrasensitive, reversible, and mechanical durable photoresponse even after being bent for 100 times. The findings suggest that SnS_1.26_Se_0.76_ can act as a promising 2D material for flexible and wearable optoelectronic devices.

## 2 Experimental

### 2.1 Growth of SnS_1.26_Se_0.76_ crystal

SnS_1.26_Se_0.76_ crystal was synthesized by reacting the pure elements in quartz ampoules *via* chemical vapor transport using iodine as transport agent.^30,31^ Specific stoichiometric uniform mixtures of Sn powder (Alfa Aesar, 99.98%), sublimed S powder (Alfa Aesar, 99.5%), and Se powder (Alfa Aesar, 99+%) were mixed in air and put into quartz tubes, and then sealed into the furnace to sinter for getting SnS_1.26_Se_0.76_ polycrystal. Subsequently, the SnS_1.26_Se_0.76_ polycrystal was mixed with iodine (density: 5 mg cm^−3^), followed by seal in the silica tubes and heating for 10–20 days in a horizontal two-zone furnace. The as-grown SnS_1.26_Se_0.76_ single crystal was transported from the hot to cold zone. Finally, the SnS_1.26_Se_0.76_ crystal was obtained by natural cooling to room temperature.

### 2.2 Structural characterization of as-synthesized SnS_1.26_Se_0.76_ crystal

The crystallographic structures of bulk SnS_1.26_Se_0.76_ were examined by X-ray diffraction (XRD, D/MAX-TTRIII (CBO)) technique. Morphologies were characterized by field emission scanning electron microscopy (FESEM, S4800,Tokyo, Japan). Specimens for transmission electron microscopy (TEM) and scanning transmission electron microscopy (STEM) observations were prepared by transferring SnS_1.26_Se_0.76_ nanosheets to the micro TEM grid. The TEM, high resolution TEM (HRTEM), selected area electron diffraction (SAED) and high-angle annular dark-field (HAADF) and bright-field (BF) STEM images were acquired by double aberration-corrected scanning transmission electron microscope (FEI G3 Cubed Themis 60–300 kV Monochromated X-FEG S/TEM). The elemental analysis were carried out by energy dispersive X-ray spectroscopy on the STEM (EDS, Super-X EDX System: four windowless Silicon Drift Detector).

### 2.3 Optical spectroscopy measurements of SnS_1.26_Se_0.76_ nanoplates

SnS_1.26_Se_0.76_ nanosheets with different thickness were mechanically exfoliated from bulk SnS_1.26_Se_0.76_ crystals onto a SiO_2_/Si substrate. Morphology and thickness of the SnS_1.26_Se_0.76_ nanosheets were obtained by a laser scanning confocal microscope (LSCM, Olympus, LEXT-OLS4000) and an atomic force microscope (AFM, Multimode 8, Bruker). The Raman mapping and spectrum were taken using a spectrometer with an excitation wavelength of 514 nm (Renishaw inVia), aimed to characterize the composition-dependent vibration modes of the synthesized SnS_1.26_Se_0.76_ alloy crystals. Micro-photoluminescence (PL) spectrum was measured with the same instrument (Renishaw inVia). Theoretical calculations of the optical bandgap and electronic band structure for the bilayer and multilayer SnS_1.26_Se_0.76_ nanosheets were carried out based upon density functional theory (DFT).

### 2.4 Fabrication and measurements of photodetector

For the device fabrication, SnS_1.26_Se_0.76_ nanosheets were obtained by mechanically exfoliating of bulk SnS_1.26_Se_0.76_ crystals onto a flexible PET substrate. TEM copper grid as a shadow mask with a typical gap of 15 μm was mounted on surface of flexible PET substrate covered by SnS_1.26_Se_0.76_ nanosheets. 10 nm Cr and 60 nm Au were subsequently evaporated as the electrodes of two-terminal photodetector, and the devices were prepared after removing the copper grid. All the electrical measurements were performed with a semiconductor test system (Agilent-B2902) at room temperature in the ambient. The 450, 532, 638 and 808 nm lasers were employed to perform photoresponse experiments.

## 3 Results and discussion

### 3.1 Structural characterization of as-synthesized SnS_1.26_Se_0.76_ crystal

**Fig. 1:**
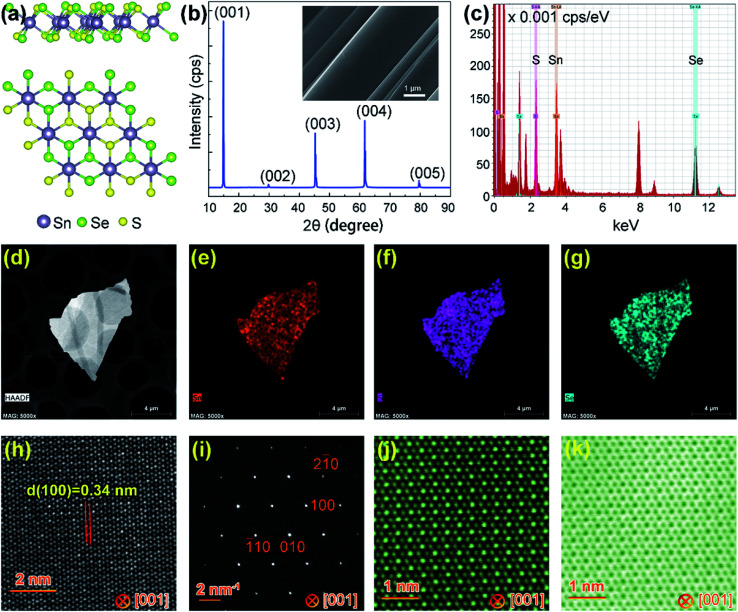
Structural characterization of as-synthesized SnS_1.26_Se_0.76_ crystal. (a) Atomic model of SnS_1.26_Se_0.76_ from a side and top view (along the [001] direction). (b) Single-crystal XRD spectrum. Inset: SEM image shows the layered structure of SnS_1.26_Se_0.76_. (c) Energy-dispersive spectroscopy (EDS) result. (d) Typical TEM image of a SnS_1.26_Se_0.76_ flake and corresponding EDS element mapping image for (e) Sn (f) S and (g) Se. (h) High-resolution TEM (HRTEM) image of SnS_1.26_Se_0.76_ viewed along [001] direction. (i) The corresponding selected area electron diffraction (SAED) pattern. (j and k) Atomic scale HAADF and BF STEM image of SnS_1.26_Se_0.76_ along [001] direction.

Bulk SnS_1.26_Se_0.76_ single crystals were synthesized by the chemical vapor transport (CVT) method with iodine as a transport agent (see Experimental). Fig. 1(a) presents the atomic structure of SnS_1.26_Se_0.76_ viewed from side and top, showing that it has a hexagonal CdI_2_-type structure with periodic S(Se)–Sn–S(Se) stacking along the *c*-axis and connecting with each layer through van der Waals forces. X-ray diffraction (XRD) of the synthesized SnS_1.26_Se_0.76_ single crystals (Fig. 1(b)) reveals five sharp peaks which correspond to (001), (002), (003), (004), and (005) peak derived from *c*-axis of the bulk, consolidating its single crystalline nature. The inset of Fig. 1(b) shows a FESEM image of SnS_1.26_Se_0.76_ single crystal exhibiting its layered structure. Further EDS analysis (Fig. 1(c)) reveals that the Sn/S/Se atomic ratio in the sample is roughly estimated to be 1 : 1.26 : 0.76, matching well with the nominal formula of SnS_2*x*_Se_2(1−*x*)_ (*x* ∼ 0.6). Fig. 1(d)–(g) shows a typical TEM image of a SnS_1.26_Se_0.76_ few-layer flake and the corresponding EDS element mapping for Sn, S and Se. The EDS mappings confirm a highly uniform distribution of Sn, S and Se elements.

Fig. 1(h) shows HRTEM image of the SnS_1.26_Se_0.76_ sample, revealing a hexagonal structured lattice with an interplanar distance of 0.34 nm, consistent with that of the (100) plane of SnS_1.26_Se_0.76_. The corresponding SAED pattern shown in Fig. 1(i) displays the lattice arrangement, indicating that the SnS_1.26_Se_0.76_ sample is stacked along [001] direction with good crystallinity. To further extract atomic information, we show in Fig. 1(j) and (k) atomic-resolution HAADF and BF STEM images of the SnS_1.26_Se_0.76_ flake viewed from the [001] axis. Since intensity of an atomic column in a HAADF STEM imaging mode is proportional to *Z*^1.7^ (*Z* is atomic number),^32–34^ the brightest spots (corresponding to the highest intensity line profile in Fig. S1(c)†) represent the Sn atomic columns (*Z* = 50), while the medium-brightness and lowest-brightness spots (corresponding to the medium–high and lowest intensity line profile in Fig. S1(c),† respectively) represent the Se-rich (*Z* = 34) and S-rich (*Z* = 16) atomic columns, respectively, as also sketched in Fig. S1(d).† Interestingly, the Se and S atoms are partially ordered and periodically distributed in the SnS_1.26_Se_0.76_ single crystal. The obtained atomic-resolution HAADF and BF STEM images (Fig. 1(k) and (j)) agree well with the atomic model (Fig. 1(a)), showing a symmetrical hexagonal lattice fringe due to the equivalent locations of Se and S in the atomic structure of SnS_1.26_Se_0.76_.

### 3.2 Optical properties of SnS_1.26_Se_0.76_ nanoplates

**Fig. 2:**
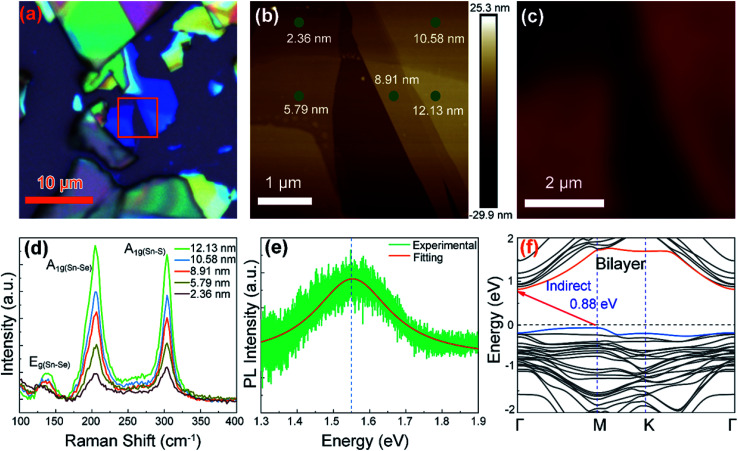
Optical properties of SnS_1.26_Se_0.76_ nanoplates deposited on SiO_2_/Si substrate. (a) Optical and (b) AFM images with different color showing different thickness. The thickness of 2.36 nm measured by AFM is about 2–3 layers. (c) Raman (200–205 cm^−1^) mapping image about the intensity of A_1g(Sn–Se)_ vibration mode measured in the same SnS_1.26_Se_0.76_ sample. (d) Raman spectrum dependent on thickness. (e) Photoluminescence spectrum and (f) calculated electronic band structure of bilayer SnS_1.26_Se_0.76_ indicating bilayer SnS_1.26_Se_0.76_ is an indirect bandgap semiconductor. The calculation also demonstrates bulk SnS_1.26_Se_0.76_ is indirect bandgap semiconductor.

The few-layer SnS_1.26_Se_0.76_ was mechanically exfoliated from SnS_1.26_Se_0.76_ crystals onto a SiO_2_/Si substrate. Fig. 2(a) shows optical images of the 2D layered SnS_1.26_Se_0.76_ with different thicknesses that can be reflected by different colors. Further AFM topography (Fig. 2(b)) reveals that the area of the palest blue color in the orange square in Fig. 2(a) has a thickness of 2.36 nm (*i.e.* 2–3 layer). To characterize the composition dependent vibration modes of the SnS_1.26_Se_0.76_ crystal, we present in Fig. 2(c) Raman mapping image for the intensity of A_1g(Sn–Se)_ vibration peak (200–205 cm^−1^). The intensity of bilayer SnS_1.26_Se_0.76_ sample is much weaker than that of few-layer one, as is also reflected in the thickness-dependent Raman spectrum (Fig. 2(d)). All the samples have characteristic peaks at about 204 and 304 cm^−1^, which are assigned to the A_1g(Sn–Se)_ and A_1g(Sn–S)_ vibration modes,^35,36^ while the weak peaks located at 136 cm^−1^ is assigned to in-plane mode E_g(Sn–Se)_. The Raman mode of E_g(Sn–S)_ is not observed possibly resulting from the weak electron–phonon interaction in thin layer samples. For the flakes with different thicknesses, the positions of these observed peaks almost remain unchanged but their intensities increase with the thickness of SnS_1.26_Se_0.76_ nanosheets. The Raman peak positions are almost the same for the samples with thicknesses ranging from 2.36 to 12.13 nm, indicating that the layer thickness has minor effect on Raman behavior.

Fig. 1(e) shows PL spectrum, where the intense peak position is located at about 1.55 eV, indicating that the optical bandgap is 1.55 eV. The intensity of PL is weaker for thicker sample due to the indirect bandgap nature of SnS_1.26_Se_0.76_, which can also be proven by calculated electronic band structure of bilayer and bulk SnS_1.26_Se_0.76_ (Fig. 2(f) and S2†). From Fig. 2(f), the bilayer SnS_1.26_Se_0.76_ is calculated to be an indirect band gap semiconductor with conduction band minimum (CBM) located at Γ point and valence band maximum (VBM) at M point. This differs from the SnS_1.26_Se_0.76_ bulk case showing an indirect band gap CBM at Γ point and VBM at a general point along M–Γ line (Fig. S2†). The bandgaps for the bilayer and bulk SnS_1.26_Se_0.76_ are estimated as 0.88 eV and 0.94 eV, respectively, which are smaller than the experimental data due to the well-known underestimation of bandgap in the hybrid functional theory (HSE06).^37^ The narrower bandgap for the bilayer with respect to its bulk implies a broad spectra detection from visible to NIR light for optoelectronic device applications.

### 3.3 Photoresponse of flexible optoelectronic devices based on SnS_1.26_Se_0.76_ nanosheets

**Fig. 3:**
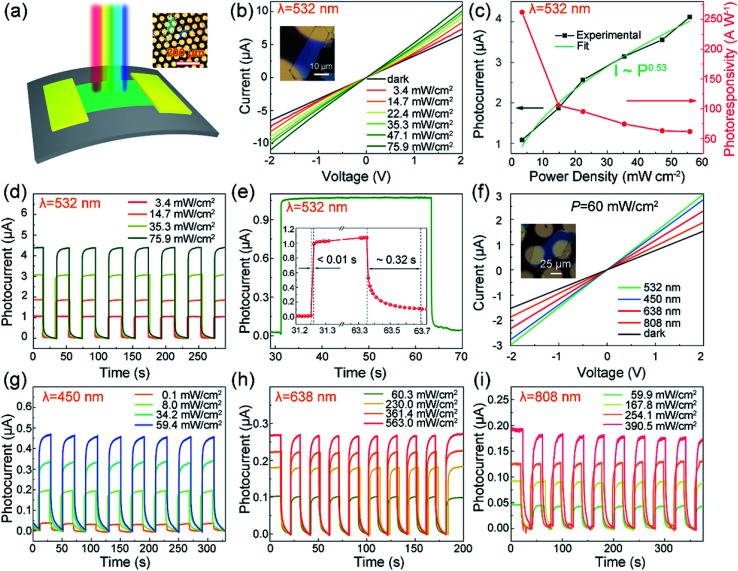
Photoresponse of flexible optoelectronic devices based on SnS_1.26_Se_0.76_ nanoplates fabricated on PET substrate. (a) Schematic illustration of the structure of a SnS_1.26_Se_0.76_ nanosheets-based two-terminal optoelectronic device; inset is the optical image of electrodes array. (b) *I*–*V* curves in the dark and under different irradiances with laser wavelength of 532 nm; inset is the optical image of the device and thickness is ∼60 nm. (c) Plots of the photocurrent and photoresponsivity against irradiance at 2 V bias (532 nm light). (d) Time-dependent photoresponse of SnS_1.26_Se_0.76_ nanoplate device at various light intensity at 2 V bias (532 nm light). (e) A separated temporal photocurrent response and reset cycle; inset is an enlarged view of the temporal photocurrent response. (f) *I*–*V* curves in the dark and in the presence of 375, 473, and 632 nm laser of a single SnS_1.26_Se_0.76_ nanoplate photodetector at a fixed irradiance of 60 mW cm^−2^; inset is the optical image of the device. (g)–(i) are time-dependent photoresponse of SnS_1.26_Se_0.76_ nanoplate device under different irradiances with laser wavelength of 450 nm, 638 nm and 808 nm, respectively. The corresponding voltage bias is 2 V.

To probe the broad spectrum optoelectronic characteristic of SnS_1.26_Se_0.76_, we fabricate flexible photodetectors based on SnS_1.26_Se_0.76_ nanopsheets on PET substrate by traditional device fabrication techniques and measure the photoresponse from visible to NIR light. Fig. 3(a) sketches the structure of a SnS_1.26_Se_0.76_ nanosheets-based two-terminal flexible photodetector (inset signifies the optical image of electrodes array). Fig. 3(b–e) exhibit high photoresponse to 532 nm light with various illumination. Fig. 3(b) shows the typical *I*–*V* curves of a device (inset of Fig. 3(b)) in the dark and under different irradiances at 532 nm. The observed linear plots of *I*–*V* indicate an ohmic contact between the SnS_1.26_Se_0.76_ film and Au/Cr electrodes. Upon decreasing the light irradiance, the photocurrent decreases, while the photoresponsivity increases (Fig. 3(c)). Here, photocurrent (*I*_ph_) is defined as the differences between *I*_illuminated_ and *I*_dark_ (*I*_ph_ = *I*_illuminated_ − *I*_dark_) and photoresponsivity (*R*_λ_) is defined as the photocurrent generated per unit power of incident light on the effective area (*S*) (*R*_λ_ = *I*_ph_/*PS*). In addition, the detectivity (D*) reflects the photodetector's sensitivity, and can be calculated by D* = *R*_λ_*S*^1/2^/(2*eI*_dark_)^1/2^, where *e* is the electronic charge. At a bias voltage of 2 V and a light irradiance of 3.4 mW cm^−2^, the device shows a photoresponsivity of 262 A W^−1^ and a detectivity of 1.98 × 10^11^ jones under the illumination of 532 nm laser. The efficient irradiation area *S* is 120 μm^2^. The corresponding light intensity dependence of the photocurrent can be expressed by a power law function of *I*_ph_ ∼ *P*^α^, which indicates increased photons generate more electrons. A power dependence of *I*_ph_ ∼ *P*^0.53^ is estimated for our photodetector by fitting the experimental data, and the deviation from the ideal slope may be attributed to the long-channel in the SnS_1.26_Se_0.76_ photodetector.

To shed light on the photoresponse with the laser on and off, we measure time-dependent photoresponse of SnS_1.26_Se_0.76_ nanosheet-based devices at various light intensity at 2 V bias (532 nm light) as shown in Fig. 3(d). With the on/off switching of incident laser, a highly stable and reversible photo-switching behavior between two states is observed and the sharp rise and drop of current with light on and off confirm that the device is ultrasensitive to light. The response time (*τ*_on_, defined as he time needed to reach 90% of the maximum photocurrent) and recovery time (*τ*_off_, defined as the time needed to drop to 10% of the maximum photocurrent) are estimated to be ∼0.01 s and ∼0.32 s, respectively, from an enlarged single circle in Fig. 3(e), which are superior to the values reported for most of layered materials.^38,39^ To evaluate the performance of our fabricated SnS_1.26_Se_0.76_ photodetectors, we list in Table 1 the outstanding figure-of-merit of some devices reported for other 2D tin dichalcogenides.^26,27,40–42^ As can be seen from Table 1, the response time in our work is very fast, but the decay time is longer than most of the previous report. The decay time of the device contains three procedures: the first stage is the recombination of excess carrier, followed by the empty of shallow and deep traps, respectively.^43^ According to the atom ratio measured by EDS, we can confirm that there are small amount of Sn vacancies appearing in SnS_1.26_Se_0.76_ crystal during the synthesis process. Therefore, the observed long decay time of the device was caused by the slow release of electrons trapped in the Sn vacancies-induced deep traps^44,45^ because the de-trap time of carriers from a deep trap can be prolonged by several orders of magnitude as compared to shallow traps, resulting in additional decay of the device.^46,47^ In addition, the SnS_1.26_Se_0.76_ shows pronounced photoresponse under a voltage bias of 2 V for 450, 532, 638, and 808 nm laser (Fig. 3(f)), and the devices show a high stability under different irradiances for different laser wavelengths (Fig. 3(d, g–i)). The SnS_1.26_Se_0.76_ photodetector exhibits the highest *R*_λ_ to 532 nm light (∼262 A W^−1^) and a relatively low *R*_λ_ to NIR light (∼120 mA W^−1^). The related performance parameters are listed in Table 2 and detailed experimental data are given in Fig. S4–S6.† As summarized in Tables 1 and 2, our photodetector shows a good photoresponse, indicating that the SnS_1.26_Se_0.76_ nanosheets can serve as an ideal 2D material as high-performance photodetectors. Further work we will focus on the study of increasing photocurrent by decreasing membrane thickness or fabricating heterojunction structure and improve experimental method to study the response to weaker light.^48,49^

**Table 1:** Comparison of outstanding figures-of-merit for photodetectors reported based on tin dichalcogenides (SnS_2_, SnSe_2_)

Materials	Synthesis method	Wavelength [nm]	*R*_λ_ [mA W^−1^]	Rise time [ms]	Decay time [ms]	D* [jones]	Ref.
SnS_2_	CVD	365	2.6 × 10^5^	20	16	10^10^	40
SnS_2_	CVD	457	8.8	5 × 10^−3^	7 × 10^−3^	10^9^	26
SnS_2_^a^	—	405	4.7 × 10^−4^	820	620	—	41
SnSe_2_	CVD	530	1.1 × 10^6^	14.5	8.1	10^10^	42
SnSe_2_	CVT	633	500	2.1	3.2	—	27
SnS_1.26_Se_0.76_^a^	CVT	532	2.62 × 10^5^	<10	320	10^11^	This work

a Flexible photodetector.

**Table 2:** Summary of the performance parameters for SnS_1.26_Se_0.76_ flexible photodetector^a^ in this work

Wavelength [nm]		Rise time [s]	Recovery time [s]	*R*_λ_ [mA W^−1^]
532	PET	<0.01	0.32	2.62 × 10^5^
	PET^b^	<0.01	0.32	2.50 × 10^5^
450	PET	1	8	11.42 × 10^3^
	PET^b^	1	8	10.01 × 10^3^
638	PET	0.45	2.4	273
	PET^b^	0.45	2.4	262
808	PET	2	2.5	120
	PET^b^	2	2.5	98

a These devices are measured under illumination of the 532 nm, 450 nm, 638 nm and 808 nm laser with a light intensity of 3.4 mW cm^−2^, 8.0 mW cm^−2^, 60.3 mW cm^−2^ and 59.9 mW cm^−2^, respectively. The corresponding voltage bias are all 2 V.

b These PET devices are measured after bending 100 times with a bending radius of 5.5 mm.

### 3.4 Durability of SnS_1.26_Se_0.76_ flexible photodetector

**Fig. 4:**
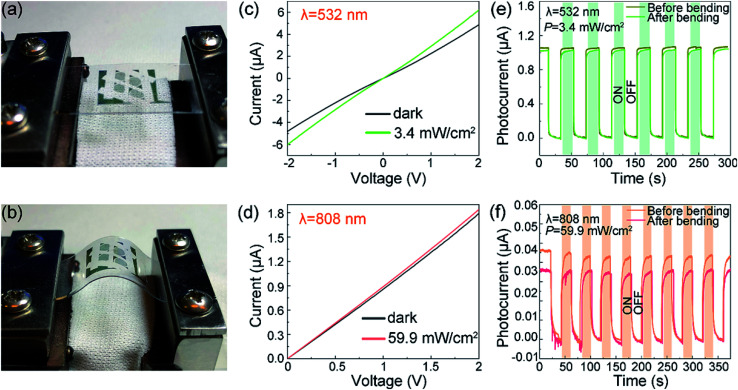
Durability measurements of SnS_1.26_Se_0.76_ flexible photodetector on PET substrate. (a) and (b) Photograph of instrument used for bending. (c) and (d) *I*–*V* curves under 532 nm laser irradiance of 3.4 mW cm^−2^ and 808 nm laser irradiance of 59.9 mW cm^−2^, respectively, after bending the device for 100 times. The black curves refer to dark current. (e) and (f) Time trace of photoresponse under illumination with 532 nm and 808 nm laser before and after bending the device for 100 times. The laser power intensity is set to 3.4 mW cm^−2^, and 59.9 mW cm^−2^, respectively. (2 V bias voltage and 5.5 mm bending radius).

Apart from the stability and sensitivity, mechanical flexibility is also an important parameter to evaluate for the flexible photodetector. In order to assess the durability of the SnS_1.26_Se_0.76_ flexible optoelectronic devices, the devices were bent repeatedly for 100 times with a bending radius of 5.5 mm, as shown in Fig. 4(a) and (b). Fig. 4(c) shows the *I*–*V* characteristics of the photodetector under a dark and 532 nm laser irradiance of 3.4 mW cm^−2^ and Fig. 4(d) under a dark and 808 nm laser irradiance of 59.9 mW cm^−2^ after bending for 100 times. The *I*–*V* plots differ slightly for both dark current and photocurrent compared with unbending case, which can be attributed to the destructive contact barrier between SnS_1.26_Se_0.76_ nanosheet and electrodes after plenty of continuous mechanical bending. Although the photocurrent has a slight decrease after bending, a sharp increase in photocurrent is observed once the devices is illuminated by either 532 nm laser or 808 nm laser, as shown in Fig. 4(e) and (f). Similar time trace of photoresponse is also observed under illumination with 450 nm and 638 nm laser, as shown in Fig. S7.† From the time-dependent photocurrent, we can see that when the laser switches from “OFF” to “ON” state, the current rapidly reaches a relatively large value and then slowly increases to a saturation value. The photocurrent decreases from ∼1.08 μA to ∼1.03 μA under 532 nm laser illumination of 3.4 mW cm^−2^ at a bias of 2 V, which leads to a minor decrease in photoresponsivity from 262 A W^−1^ to 250 A W^−1^. However, the response time and recovery time remain almost unchanged after sustaining 100 times of bending. The photoresponse parameters of *τ*_on_, *τ*_off_ and *R*_λ_ are summarized in Table 2 before and after bending 100 times with different illumination wavelength. These findings show that SnS_1.26_Se_0.76_ nanosheet-based photodetector maintains excellent mechanical durability, reproducibility, and high photo-sensitivity, demonstrating great potential for advanced flexible and wearable optoelectronic device applications.

## 4 Conclusions

We perform a systematic study on flexible photodetectors based on high-quality single crystalline ternary SnS_1.26_Se_0.76_ nanosheets. The photodetectors exhibit a good broad spectra photoresponse in the region of visible (450 nm) to NIR (808 nm) light, high photoresponsivity of ∼262 A W^−1^, and fast response time of ∼10 ms to 532 nm light. Furthermore, such photodetector can maintain excellent mechanical durability, reproducibility, and high photo-sensitivity even after being bent for 100 times with a bending radius of 5.5 mm, showing great potential in the application of advanced flexible and wearable optoelectronic devices. The findings pay a new way in employing other layered alloy materials in flexible optoelectronic devices based on bandgap engineering.

## Author contributions

C. W. and Q. L. approached this idea. L. N. D. and C. W. contributed equally to this work. Q. L. and Cong Wang guided this research. L. N. D. and C. W. worked on device fabrication, performed the measurements and analyzed the data. L. N. D., C. W. and Q. L. wrote the manuscript. W. Q. X. performed electronic structure calculations. Z. C. W., Z. M. W., X. Z. Z. and J. B. L. made suggestions to improve this research. B. W. measured STEM and EDS. X. F. W. plotted the schematic illustration of SnS_1.26_Se_0.76_ flexible photodetector. We thank J. Z. F., X. T. W. and L. J. M. for their help in electrical measurements. All authors discussed experimental and theoretical results.

## Conflicts of interest

There are no conflicts to declare.

## Supplementary Material

RA-009-C9RA01734H-s001
