# Supplementary material for: A ternary SnS1.26Se0.76 alloy for flexible broadband photodetectors
Source: RSC Adv. 2019 May 7;9(25):14352–9. doi: 10.1039/c9ra01734h (PMC9064034; doi:10.1039/c9ra01734h)
Supplement: RA-009-C9RA01734H-s001 [file RA-009-C9RA01734H-s001.pdf]

Electronic Supplementary Material (ESI) for RSC Advances.
This journal is © The Royal Society of Chemistry 2019

A ternary $SnS_{1.26}Se_{0.76}$ alloy for flexible broadband photodetectors

Lena Du,‡[a] Cong Wang,‡[a,b] Jingzhi Fang,[c] Bin Wei,[d] Wenqi Xiong,[e] Xiaoting Wang,[c] Lijun Ma,[a] Xiaofeng Wang,[a] Zhongming Wei,[c] Congxin Xia,[e] Jingbo Li,*[c] Zhongchang Wang,*[d] Xinzheng Zhang,*[b] and Qian Liu*[a,b,c]

[a]*CAS Key Laboratory of Nanosystem and Hierarchical Fabrication, CAS Center for Excellence in Nanoscience, National Center for Nanoscience and Technology, Beijing 100190, P. R. China, University of Chinese Academy of Sciences, Beijing 100049, P. R. China. E-mail:liuq@nanoctr.cn*

[b]*The MOE Key Laboratory of Weak-Light Nonlinear Photonics, TEDA Institute of Applied Physics, Nankai University, Tianjin 300457, P. R. China. E-mail: zxz@nankai.edu.cn*

[c]*State Key Laboratory of Superlattices and Microstructures, Institute of Semiconductors, Chinese Academy of Sciences, Beijing 100083, P. R. China. E-mail: jbli@semi.ac.cn*

[d]*Department of Quantum and Energy Materials, International Iberian Nanotechnology Laboratory (INL), Avenida Mestre José Veiga s/n, Braga 4715-330, Portugal. E-mali: zhongchang.wang@inl.int*

[e]*Department of Physics, Henan Normal University, Xinxiang 453007, P. R. China.*

‡These authors contributed equally.

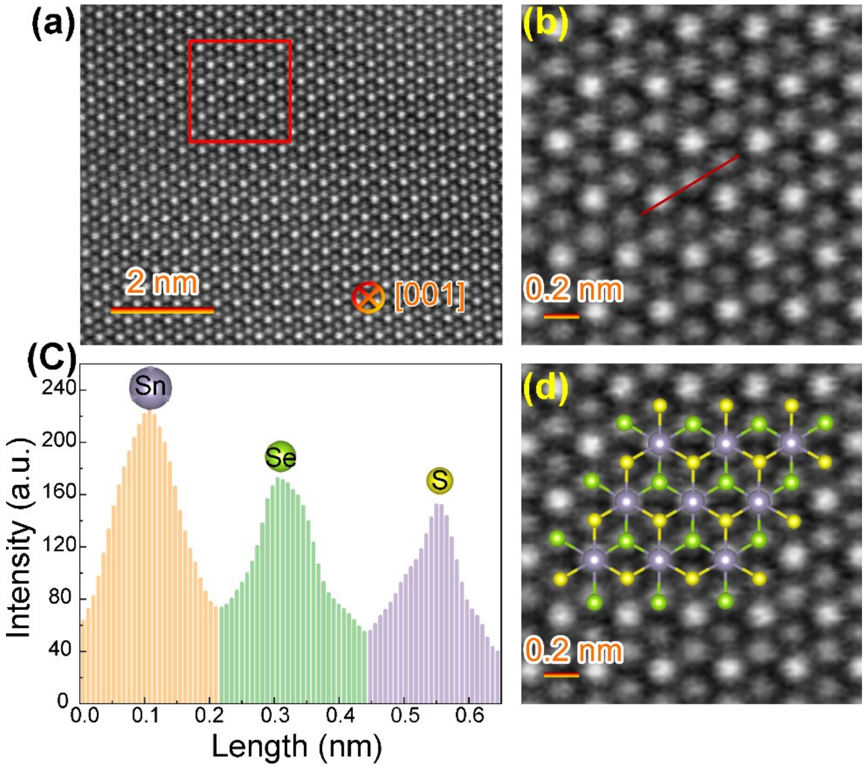

Figure S1. Atom-by-atom analysis in $SnS_{1.26}Se_{0.76}$ by HAADF STEM. (a) Atomic scale HAADF STEM image of $SnS_{1.26}Se_{0.76}$ along [001] direction. (b) Atom-resolved higher magnification HAADF image of the region for red rectangle marked in (a). (c)The experimental image intensity line profiles obtained along red line in panel (b). The intensity profile shows three levels of brightness representing different atoms for Sn, Se, and S in the $SnS_{1.26}Se_{0.76}$ alloy. (d) Structure model obtained from intensity analysis showing the distribution of Sn, S, Se atoms.

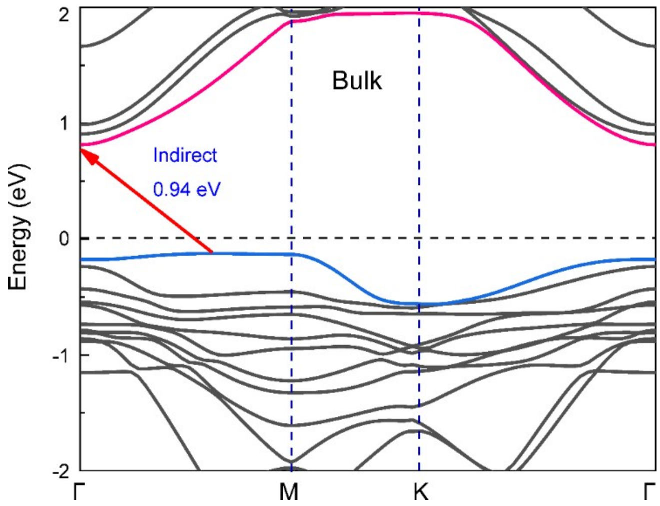

Figure S2. Calculated electronic band structure of bulk $SnS_{1.26}Se_{0.76}$ indicating bulk $SnS_{1.26}Se_{0.76}$ is an indirect bandgap semiconductor with a bandgap of 0.94 eV.

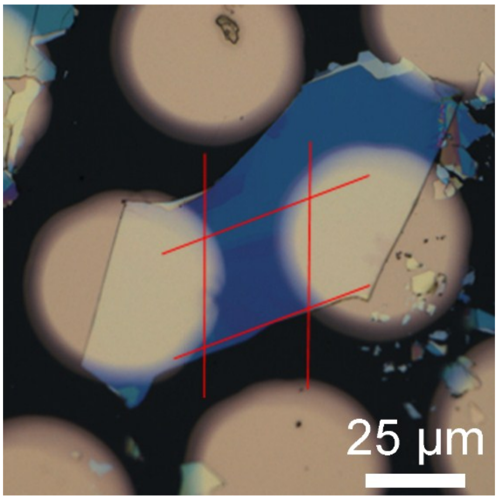

Figure S3. An optical image of the device.

As can be seen from Figure S3, the $SnS_{1.26}Se_{0.76}$ nanosheet is not very uniform, since the size and shape of the $SnS_{1.26}Se_{0.76}$ nanosheets are uncontrollable using mechanical exfoliation. Here, we mainly focus on the study of the performance of PDs, and thus relative to seeking the sample being as uniform as possible, we pay more attention to the good performance of the devices.

It should be noticed that, in this work, we mainly study the broad spectrum photoresponse of $SnS_{1.26}Se_{0.76}$ photodetectors and their respective characteristic under different wavelength lasers with various power densities, so we only noticed to measure the similar thickness samples and ignored the identity of the samples used. We test different devices to optimize the performance of photodetectors under 532 nm wavelength lasers with different irradiances.

As for the calculation of effective area, to ensure maximum effective areas are included, the closed region within the red line in Figure S3 was calculated. The effective area is 25 μm$\times$24μm = 600 μm^2.

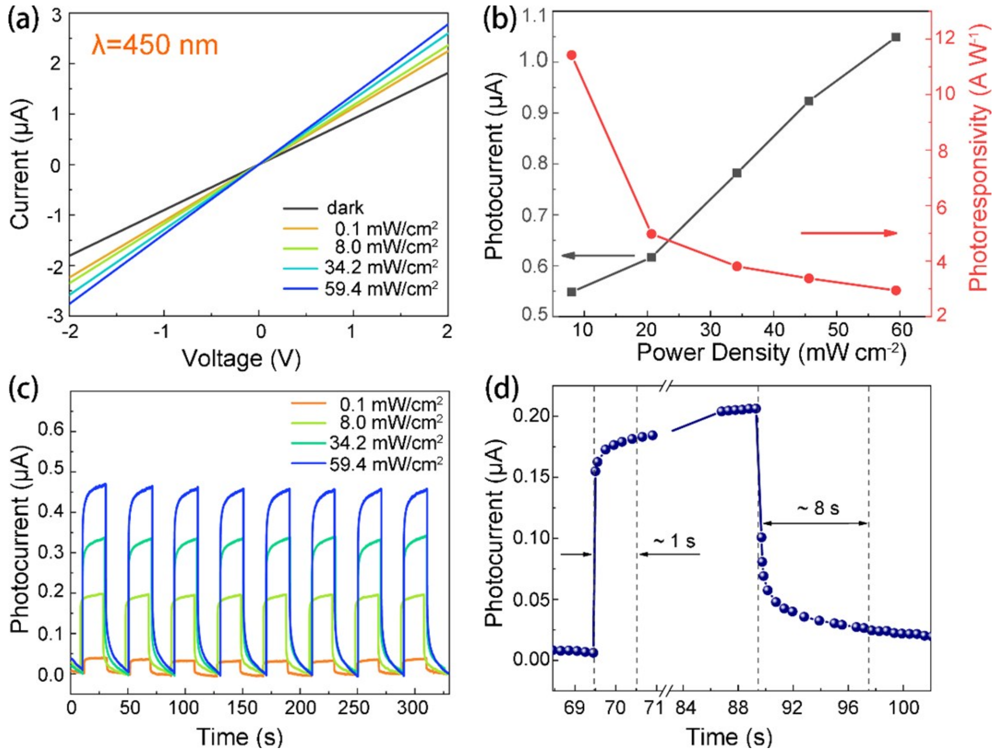

Figure S4. Photoresponse of flexible optoelectronic devices based on $SnS_{1.26}Se_{0.76}$ nanoplates fabricated on PET substrate under 450 nm light. (a) I–V curves in the dark and under different irradiances. (b) Plots of the photocurrent and photoresponsivity against irradiance. (c) Time-dependent photoresponse of $SnS_{1.26}Se_{0.76}$ nanoplates-based device at various light intensity. (d) An enlarged view of the temporal photocurrent response. The corresponding voltage bias is 2V.

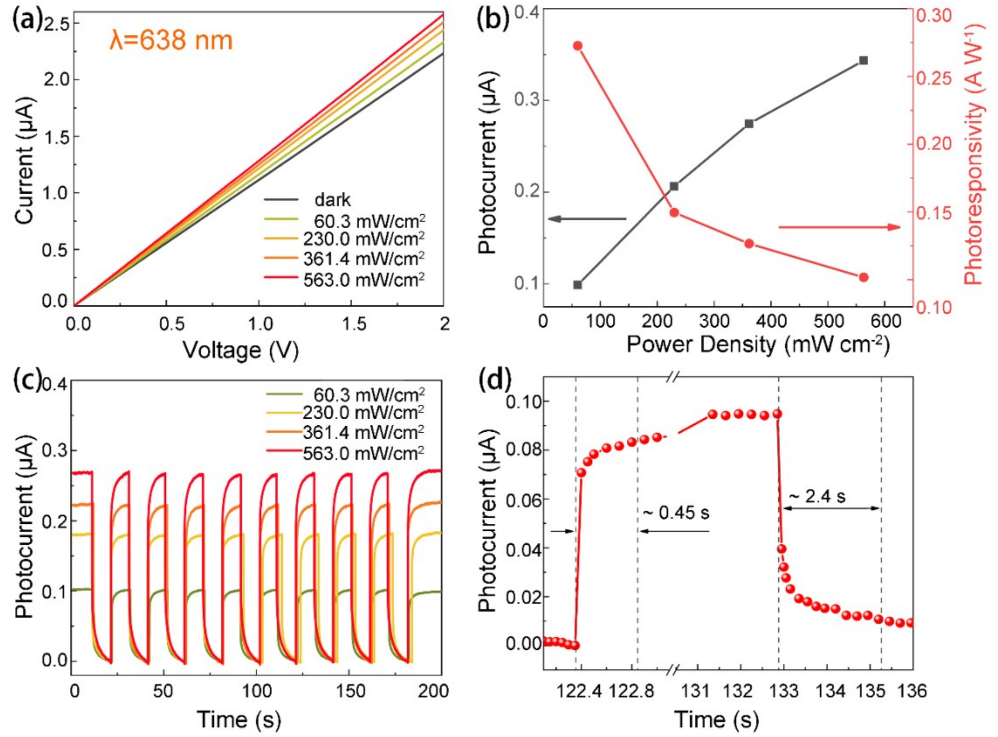

Figure S5. Photoresponse of flexible optoelectronic devices based on $SnS_{1.26}Se_{0.76}$ nanoplates fabricated on PET substrate under 638 nm light. (a) I–V curves in the dark and under different irradiances. (b) Plots of the photocurrent and photoresponsivity against irradiance. (c) Time-dependent photoresponse of $SnS_{1.26}Se_{0.76}$ nanoplates-based device at various light intensity. (d) An enlarged view of the temporal photocurrent response. The corresponding voltage bias is 2V.

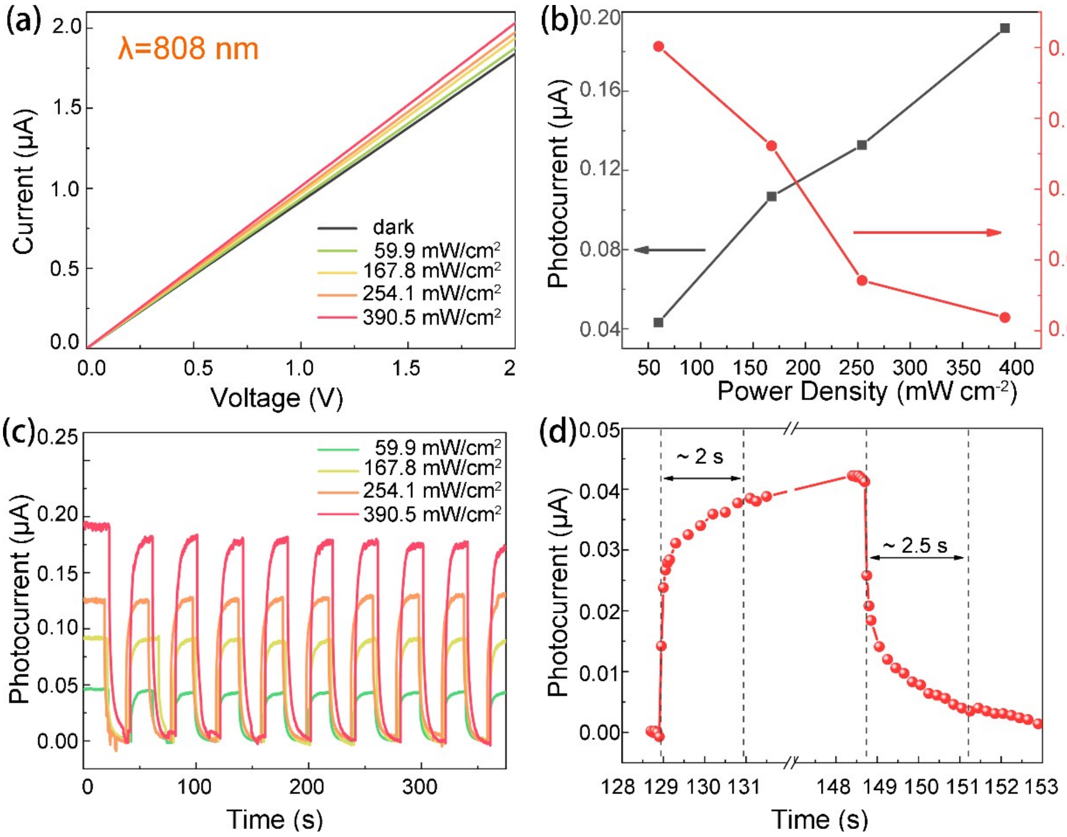

Figure S6. Photoresponse of flexible optoelectronic devices based on $SnS_{1.26}Se_{0.76}$ nanoplates fabricated on PET substrate under 808 nm light. (a) I–V curves in the dark and under different irradiances. (b) Plots of the photocurrent and photoresponsivity against irradiance. (c) Time-dependent photoresponse of $SnS_{1.26}Se_{0.76}$ nanoplates-based device at various light intensity. (d) An enlarged view of the temporal photocurrent response. The corresponding voltage bias is 2V.

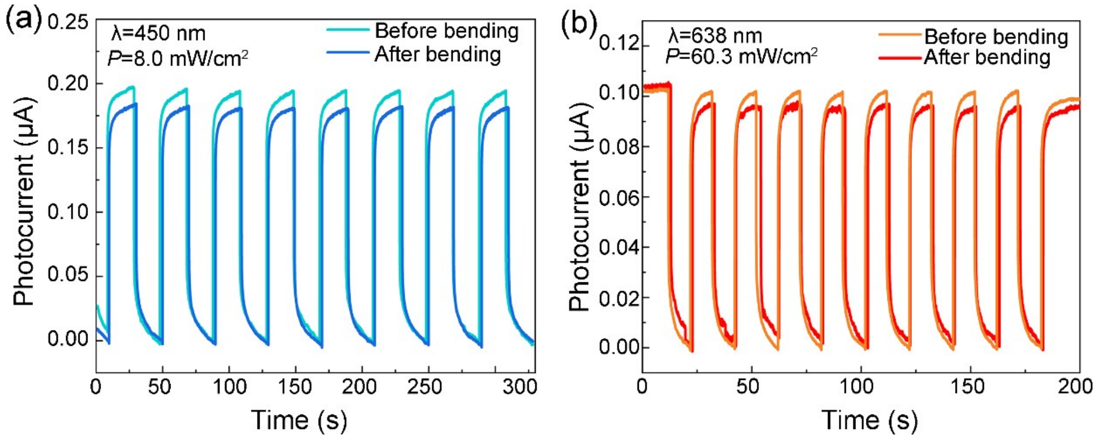

Figure S7. Durability measurements of $SnS_{1.26}Se_{0.76}$ flexible photodetector on PET substrate. (a) and (b) Time trace of photoresponse under illumination with 450 nm and 638 nm laser before and after bending the device for 100 times. The light power intensity is set to 8.0 mW/cm^2, and 60.3 mW/cm^2, respectively. (2 V bias voltage and 5.5 mm bending radius).
